# Modelling and Simulation of the Combined Use of IABP and Impella as a Rescue Procedure in Cardiogenic Shock: An Alternative for Non-Transplant Centres?

**DOI:** 10.3390/bioengineering10121434

**Published:** 2023-12-17

**Authors:** Beatrice De Lazzari, Massimo Capoccia, Roberto Badagliacca, Claudio De Lazzari

**Affiliations:** 1 Faculty of Electrical Engineering, Mathematics and Computer Science (EEMCS), Biomedical Signals and Systems (BSS), University of Twente, Drienerlolaan 5, 7522 NB Enschede, The Netherlands; 2 Northern General Hospital, Sheffield Teaching Hospitals NHS Foundation Trust, Sheffield S5 7AU, UK; capoccia@doctors.org.uk; 3 Department of Biomedical Engineering, University of Strathclyde, Glasgow G4 0NW, UK; 4 Department of Clinical, Internal Anesthesiology and Cardiovascular Sciences, “Sapienza” University of Rome, 00147 Rome, Italy; roberto.badagliazza@uniroma1.it; 5 National Research Council, Institute of Clinical Physiology (IFC-CNR), 00185 Rome, Italy; claudio.delazzari@ifc.cnr.it; 6 Faculty of Medicine, Teaching University Geomedi, 0114 Tbilisi, Georgia

**Keywords:** lumped-parameter model, CARDIOSIM©, IABP, Impella, cardiovascular system, heart failure, clinician, numerical simulator, e-learning

## Abstract

The treatment of critically ill patients remains an evolving and controversial issue. Mechanical circulatory support through a percutaneous approach for the management of cardiogenic shock has taken place in recent years. The combined use of IABP and the Impella 2.5 device may have a role to play for this group of patients. A simulation approach may help with a quantitative assessment of the achievable degree of assistance. In this paper, we analyse the interaction between the Impella 2.5 pump applied as the LVAD and IABP using the numerical simulator of the cardiovascular system developed in our laboratory. Starting with pathological conditions reproduced using research data, the simulations were performed by setting different rotational speeds for the LVAD and driving the IABP in full mode (1:1) or partial mode (1:2, 1:3 and 1:4). The effects induced by drug administration during the assistance were also simulated. The haemodynamic parameters under investigation were aa follows: mean aortic pressure, systolic and diastolic aortic pressure, mean pulmonary arterial pressure, mean left and right atrial pressure, cardiac output, cardiac index, left and right ventricular end-systolic volume, left ventricular end-diastolic volume and mean coronary blood flow. The energetic variables considered in this study were as follows: left and right ventricular external work and left and right atrial pressure-volume area. The outcome of our simulations shows that the combined use of IABP and Impella 2.5 achieves adequate support in the acute phase of cardiogenic shock compared to each standalone device. This would allow further stabilisation and transfer to a transplant centre should the escalation of treatment be required.

## 1. Introduction

The treatment of critically ill patients remains an evolving and controversial issue. Technological progress has made various mechanical circulatory assist devices (MCADs) available for the treatment of this group of patients. Some of these devices provide haemodynamic support to a dysfunctional left ventricle through a minimally invasive implantation approach using percutaneous techniques [1,2,3,4,5,6,7]. This technological evolution allows the use of MCADs in patients with haemodynamic instability, where the speed and safety of the implant is critical, as well as in more stable patients undergoing cardiovascular interventions that are potentially associated with the risk of destabilisation. The choice of the appropriate percutaneous ventricular assist device (PVAD) system, the timing of the implant, the duration of support and the prevention of any complications are the key points in the management of patients requiring the insertion of MCADs [8]. Nevertheless, the scientific evidence remains controversial and based on the experience of each centre, leading to significant variability in indications, recommendations and implantation techniques [9,10]. Despite this variability, PVAD implants in the catheterisation laboratory has taken place with promising implications for the future.

There are many studies in the literature on the use of combined peripheral venoarterial extracorporeal membrane oxygenation (VA-ECMO) and Impella 2.5 or VA-ECMO and the intra-aortic balloon pump (IABP) for the management of patients in cardiogenic shock (CS) [2,4,5,9]. More recent experimental and clinical studies focused their attention on the combined use of Impella 2.5 and IABP in CS patients [1,11,12,13,14,15,16]. Although the results presented are controversial [17,18], it is worth exploring this aspect further and determining its true potential as a prelude to a more definitive application on a routine basis.

In the study of the interaction between the cardiovascular and respiratory systems and different types of mechanical circulatory and respiratory assistance devices, numerical and hybrid simulators have proven to be excellent tools for the analysis and understanding of the effects induced by different modes of support on haemodynamic and energetic variables [4,5,19,20,21,22,23,24,25].

The purpose of this study was to explore the use of the CARDIOSIM© software (version 8.2.1) platform [19,26] to simulate the effects induced by the simultaneous administration of drugs, the activation of the IABP and the left ventricular assist device (LVAD), Impella 2.5 (Abiomed Europe GmbH, Aachen, Germany), on cardiovascular haemodynamic and energetic variables [4]. The features of CARDIOSIM© allow IABP support to be driven from full (1:1) to partial (1:2, 1:3 and 1:4). This study consisted of a virtual CS patient [4,27,28] whose baseline status was followed by the simultaneous administration of Milrinone to simulate the decrease limit only in pulmonary vascular resistances of about 10% and the activation of IABP. In addition, the simultaneous activation of IABP (without drug administration) and Impella 2.5 was addressed. The full range of IABP setting (1:1, 1:2, 1:3 and 1:4) was considered during the drug administration [29]. Here, we further develop aspects initially addressed in a previous study by our group [30].

## 2. Materials and Methods

This study was designed and based on the use of CARDIOSIM©, which is a numerical simulator of the cardiovascular system developed in the Cardiovascular Numerical/Hybrid Modelling Lab (Rome) at the Institute of Clinical Physiology (IFC-CNR), in collaboration with the Department of Human Movement and Sport Sciences, “Foro Italico” 4th University of Rome, the Department of Biomedical Engineering, University of Strathclyde (Glasgow), the Department of Clinical, Internal Anesthesiology and Cardiovascular Sciences, “Sapienza” University of Rome, and the Faculty of Medicine of Teaching University Geomedi [4,5,19,21,26,31]. The software simulator consists of lumped-parameter models of the circulatory network and the time-varying elastance concept to reproduce native ventricular, atrial and septal behaviour [21,31].

The cardiovascular network between the left ventricular output and the right atrial input, developed with 0-D models, described in [5,26], consists of the following: ascending and descending aorta, aortic arch, thoracic, abdominal, upper limbs and head, renal, hepatic, splanchnic, superior, inferior and abdominal vena cava and leg sections, respectively. The circulatory network between the right ventricular output and the left atrial input, described in [26,32], was developed using the main and small arterial, pulmonary arteriole and capillary and pulmonary venous compartments.

The 0-D model described in [4,26,31,32] simulates the coronary circulation.

The numerical models of the IABP and the Impella 2.5 device implemented on CARDIOSIM© platform were modified in order to be activated simultaneously. The IABP was operated by adjusting the vacuum pressure to –10 mmHg and drive pressure to 260 mmHg, as described in [4]. For the purposes of this study, the progressive timing reduction from full (1:1) to partial (1:2, 1:3 and 1:4) assistance was implemented for the IABP [4,30].

The Impella 2.5 (Abiomed Europe GmbH, Aachen, Germany) pump was implemented on the software platform as described in [4].

The first part of the study reproduced a virtual CS patient with its baseline status according to research data [4,33,34,35,36,37], as follows: systolic aortic pressure (SAP <90 mmHg), cardiac index (CI lower than 2.2 L/min/m^2^), pulmonary capillary wedge pressure higher than 15 mmHg, and elevated left ventricular end-diastolic pressure (LVEDP >18 mmHg).

The second part of the study focused on the simulation settings with IABP support in full mode and simultaneous drug administration (Milrinone) to reduce the pulmonary vascular resistance only, by 10% [34]. Subsequently, the weaning of the virtual patient was simulated by activating the IABP in 1:2, 1:3 and 1:4 modes. When the IABP operated in 1:2, or 1:3 or 1:4 mode, the relevant values were calculated as average of twenty-four cardiac cycles, respectively. In the last part of the study, IABP and Impella 2.5 pump were applied simultaneously with different rotational speeds of 35,000, 40,000 and 50,000 rpm for the Impella device and full (1:1) to partial (1:2, 1:3 and 1:4) assistance for the IABP. Drug administration was not considered.

The haemodynamic parameters under investigation were as follows: mean aortic pressure (AoP), systolic and diastolic aortic pressure (SAP and DAP), mean pulmonary arterial pressure (PAP), mean left atrial pressure (LAP), mean right atrial pressure (RAP), cardiac output (CO), cardiac index (CI), left and right ventricular end-systolic volume (LVESV and RVESV), left ventricular end-diastolic volume (LVEDV) and mean coronary blood flow (CBF). The energetic variables considered in the study were as follows: left and right ventricular external work (LVEW and RVEW) and left and right atrial pressure-volume area (LAPVA and RAPVA) [5].

## 3. Results

**Figure 1:**
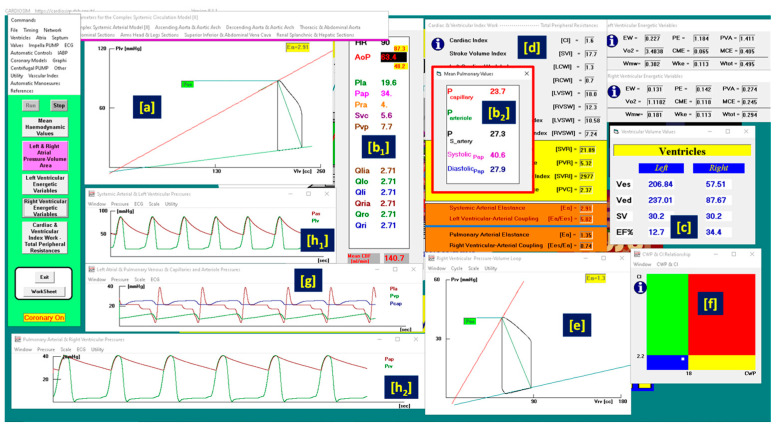
Virtual CS patient developed according to literature data. Panels (**a**,**e**) show the left and right ventricular loops, respectively; panels (**h_1_**,**h_2_**,**g**) show the instantaneous waveforms of aortic (Pas) and left ventricular (Plv) pressures, pulmonary capillary, (Pcap) pulmonary venous (Pvp) and left atrial pressures (Pla), respectively, and pulmonary arterial (Pap) and right ventricular pressures (Pra). Panels (**b_1_**,**b_2_**) show the mean values of pressures and flows (AoP≡Pas is the mean aortic pressure, 87.3 is the systolic aortic pressure (SAP) and 48.2 is the diastolic aortic pressure (DAP); CBF is the mean coronary blood flow). Panel (**c**) shows the left and right ventricular end-systolic and end-diastolic volumes, the stroke volume and the ejection fraction. Panel (**d**) shows the CI, systemic (pulmonary) arterial elastance and left (right) ventricular-arterial coupling. Finally, panel (**f**) reproduces the cardiac index (CI) and capillary wedge pressure (CWP) relationship.

A virtual CS patient was developed according to research data [4,33,34,35,36,37] (Figure 1) using CARDIOSIM© (8.2.1) software.

Figure 1 shows that the systolic aortic pressure is lower than 90 mmHg, the cardiac index is lower than 2.2 L/min/m^2^ (panel d), the pulmonary capillary wedge pressure is 23.7 mmHg and the left ventricular end-diastolic pressure is higher than 18 mmHg. The relationship graph of the cardiac index (CI) and capillary wedge pressure (CWP) (panel (f)) shows the four different regions, describing the following:

- the normal haemodynamic condition (green rectangle);
- cardiorespiratory disease induced by insufficient CO (red rectangle);
- hypoperfusion state caused by fluid-volume depletion (blue rectangle);
- low flow rate (yellow rectangle) requiring either drug therapy or IABP device, or ventricular assist device (VAD) support.

The simulated CS patient’s conditions show that the CI–CWP relationship presents a yellow point in the blue region (panel (f)), i.e., the hypoperfusion state caused by fluid-volume depletion.

**Figure 2:**
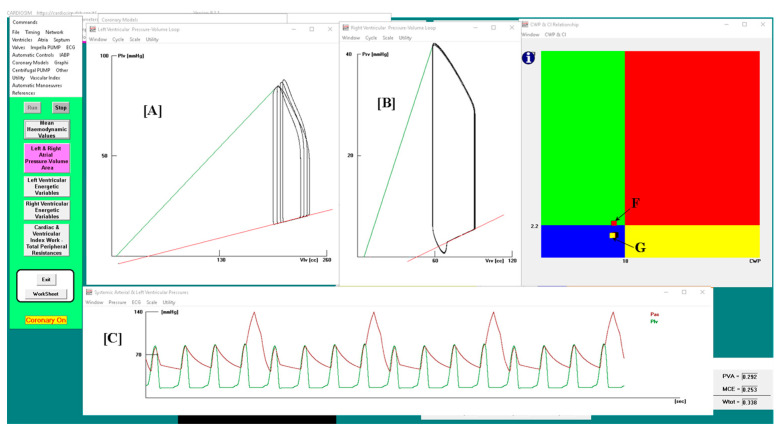
Panel (**A**,**B**) shows the left (right) ventricular pressure-volume loops. Plv≡LVP; Vlv≡LVV (left ventricular volume); Prv≡RVP; Vrv≡RVV (right ventricular volume); Pas≡AoP. Sixteen aortic and left ventricular pressure waveforms are plotted in panel (**C**). In this case, the IABP was driven in 1:4 mode and drug administration was simulated by reducing the pulmonary vascular resistances by 10%. On CI–CWP graph, the rectangle point moves from G position (IABP off) to F position (IABP on).

Figure 2 shows the results obtained when drug administration and IABP support were applied simultaneously. In this case, the IABP was driven in 1:4 mode. The left and right ventricular pressure-volume loops are plotted (in panel A and B, respectively) for four cardiac cycles: three cycles are without assistance (IABP off) and one cycle is with assistance (IABP on).

Panel C shows sixteen aortic and left ventricular pressure waveforms. After three unassisted waveforms, one assisted waveform follows. Finally, the point representing the relationship between the two parameters moves from position G (in the blue rectangle) to position F (in the green rectangle) on the CI–CWP graph.

**Figure 3:**
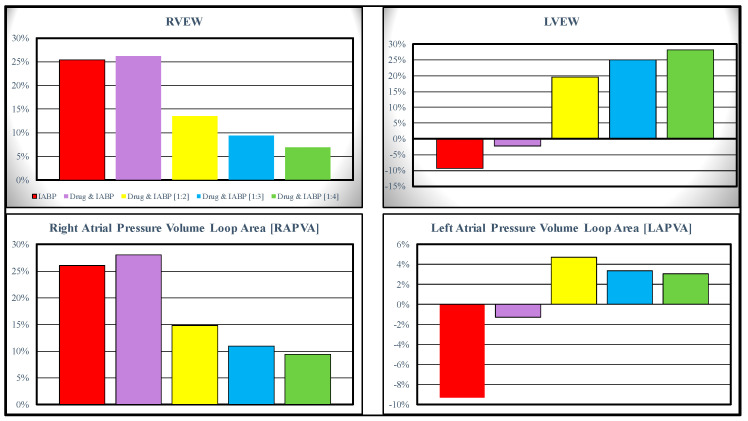
Data representing the percentage changes in relation to virtual pathological (CS) condition. The red columns represent the percentage changes when IABP was activated in full mode (1:1). The purple, yellow, light blue and green columns show the percent changes when the effects of the drug administration and IABP (driven in partial mode) are simulated simultaneously. When the IABP operates in 1:2, or 1:3 or 1:4 mode, the values presented in this paper are calculated as average values of twenty-four cardiac cycles, respectively.

Starting from baseline conditions, the effects induced on the RVEW, LVEW, RAPVA and LAPVA by the IABP activation in full and partial modes are represented in Figure 3. The top-left panel shows that the IABP (driven in full mode) without and with drug administration increased the RVEW by 25% compared to the baseline conditions. The simultaneous simulation of drug administration and IABP driven in partial (1:4) mode gave a 6% increase. The IABP support (full mode) decreased the LVEW regardless of the drug administration. The intra-aortic balloon pump increased LVEW when driven in partial mode (top-right panel).

**Figure 4:**
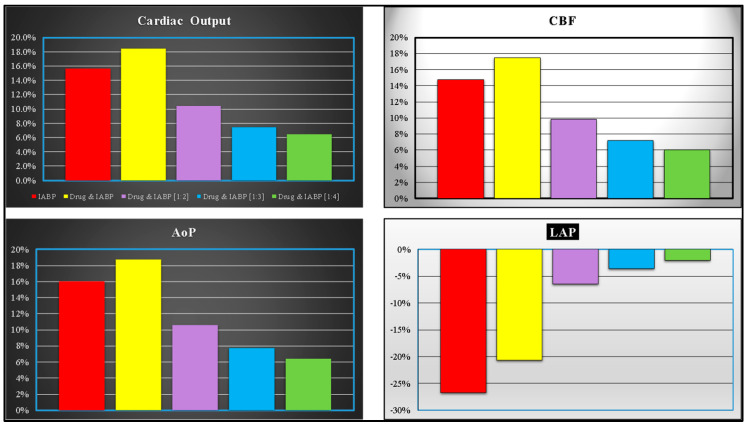
The data represent the percentage changes in relation to virtual pathological (CS) condition. The red columns represent the percentage changes when IABP was activated in full mode (1:1). The purple, yellow, light blue and green columns show the percentage changes when the effects of the drug administration and IABP (driven in partial mode) were simulated simultaneously. The percent changes of CO (coronary blood flow) are reported in the top-left (-right) panel. The bottom-left (-right) panel shows the mean aortic pressure (left atrial pressure).

The IABP support increased the RAPVA regardless of the drug administration (bottom-left panel), while different effects were induced on the LAPVA (bottom-right panel). The haemodynamic effects induced by different IABP settings and drug administrations are reported in Figure 4.

Simulation of simultaneous drug administration and IABP driven in full and partial (1:2) modes showed an increase of more than 15% (16%) in the CO (AoP) according to the research data (top- and bottom-left panels). A reduction of more than 20% was recorded for the left atrial pressure (bottom-right panel). Finally, an increase in the coronary blood flow of between 6% and 17% was obtained for all the IABP actuation modes (top left panel).

**Figure 5:**
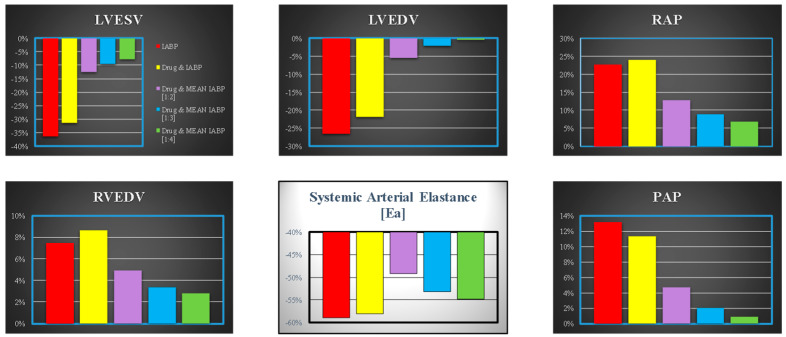
Data represent the percentage changes in relation to virtual pathological (CS) condition. The red columns represent the percentage changes when IABP is driven in full mode (1:1). The purple, yellow, light blue and green columns show the percentage changes when the effects of drug administration and IABP support (driven in 1:2, 1:3 and 1:4 mode) are simulated simultaneously. When the IABP operates in 1:2, or 1:3 or 1:4 mode, the values are calculated as average values of twenty-four cardiac cycles, respectively.

Figure 5 shows the effects induced on the left ventricular end-systolic volume (top-left panel), left ventricular end-diastolic volume (top-middle panel), mean right atrial pressure (top-right panel), mean pulmonary arterial pressure (bottom-right panel), systemic arterial elastance (bottom-middle panel) and right ventricular end-diastolic volume (bottom-left panel).

**Figure 6:**
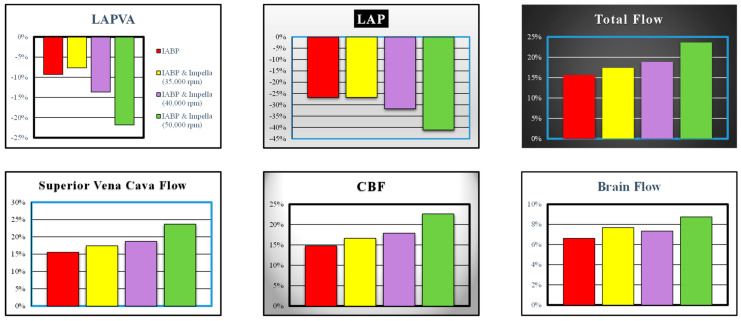
Effects induced by simultaneous application of IABP driven in full mode (1:1) and Impella 2.5 (driven with rotational speed set to 35,000, 40,000 and 50,000 rpm) on left atrial pressure-volume loop area (top-left panel), left atrial pressure (top-middle panel), superior vena cava flow (bottom-left panel) coronary blood flow (bottom-middle panel) and brain flow (bottom-right panel). The effects on total flow (i.e., cardiac natural flow pus Impella flow) are represented in the top-right panel.

The following are the effects induced on the haemodynamic and energetic variables by the simultaneous activation of the IABP and the Impella 2.5. The drug administration was not simulated to avoid interference with the simultaneous activation of the two support systems. The effects induced by the simultaneous activation of the IABP in full mode (1:1) and the Impella 2.5 pump on the left atrial pressure, left atrial pressure-volume area (LAPVA), total flow, superior vena cava flow, coronary blood flow and cerebral flow are plotted in Figure 6. The LVAD was activated with different rotational speeds (i.e., 35,000, 40,000 and 50,000 rpm). The simultaneous activation of the two devices induced a reduction of approximately 22% in the LAPVA, a 25% increase in the total flow and an increase of approximately 25% in the CBF when the rotational pump’s speed was set to 50,000 rpm. The increases and reductions were calculated with respect to the values measured in pathological conditions. The superior vena cava and brain blood flow showed increases of approximately 15% and 6%, respectively, when the IABP alone was driven in full mode.

**Figure 7:**
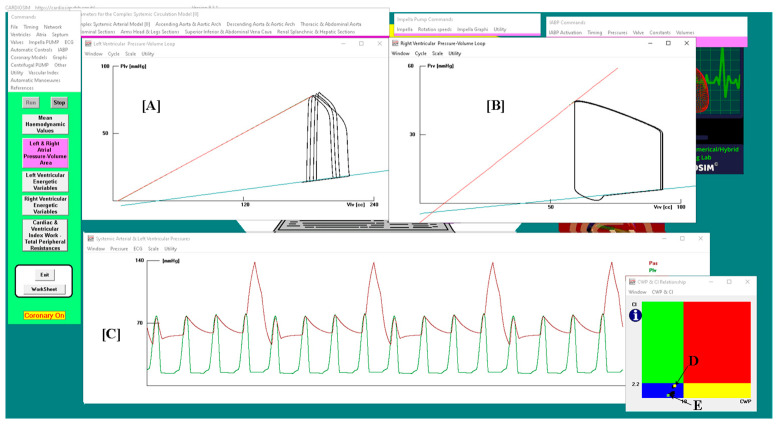
Panel (**A**,**B**) shows the left (right) ventricular pressure-volume loop. Plv≡LVP; Vlv≡LVV (left ventricular volume); Prv≡RVP; Vrv≡RVV (right ventricular volume); Pas≡AoP. Sixteen aortic and left ventricular pressure waveforms are plotted in panel (**C**). In this case, both IABP driven in 1:4 mode and Impella 2.5 with rotational speed of 40,000 rpm were applied simultaneously. The rectangle point moves from E position (IABP off) to D position (IABP on) on the CI– CWP graph.

Figure 7 shows the results obtained when both the IABP driven in the 1:4 mode and the Impella 2.5 with a rotational speed of 40,000 rpm were applied simultaneously. Four cardiac cycles are plotted in the left (A) and right (B) ventricular pressure-volume planes: three cycles without assistance (IABP off) and one cycle with assistance (IABP on).

**Figure 8:**
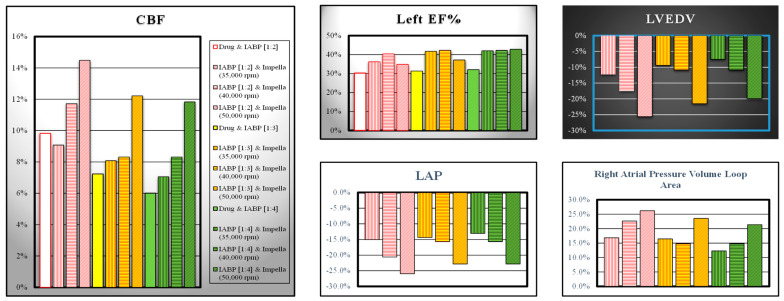
White, yellow and green bars representing the effects induced by IABP driven in 1:2, 1:3 and 1:4, respectively, when drug administration simulated is available in the left and top-middle panels on graphs. Data represent the percentage changes in relation to virtual pathological (CS) condition. The bars with vertical (horizontal) lines indicate the percentage change of a variable when the LVAD rotational speed was set to 35,000 (40,000) rpm, while the bars with oblique lines were obtained for a rotational speed of 50,000 rpm. The simulations were performed setting the IABP in 1:2 (white bars with red lines), 1:3 (yellow bars with red lines) and 1:4 (green bars with black lines) mode.

The comparison between the effects induced on some of the haemodynamic variables by the simultaneous activation of the IABP (driven in full and partial modes) and the Impella 2.5 pump (activated with different rotation speeds, i.e., 35,000, 40,000 and 50,000 rpm) is shown in Figure 8. The simulations performed in this study highlighted that for the coronary blood flow, the assistance provided with the IABP driven in full and partial modes and the drug administration produced similar effects to those induced by the simultaneous activation of the IABP (in full and partial modes) and the Impella 2.5 with a rotational speed of 35,000 rpm (white bar and bar with vertical red line in the left panel in Figure 8).

In contrast, different effects were observed on the left ventricular ejection fraction (the white bar and the bar with the vertical red line in the top-middle panel), where the simultaneous application of the IABP and Impella 2.5 generated higher percentage variations compared to the application of the IABP (driven in full and partial modes) and the drug administration (Figure 8). The top-right panel (bottom-middle panel) shows that the combination of the IABP (driven in partial mode) and the LVAD (with a rotational speed of 50,000 rpm) induced a reduction of between 25.6% and 19.88% (25.95% and 22.84%) in the left ventricular end-diastolic volume (left atrial pressure). Finally, the same combination of devices (driven in the same conditions) produced an increase of between 26.2% and 21.35% in the right atrial pressure-volume loop area (bottom-right panel in Figure 8).

**Figure 9:**
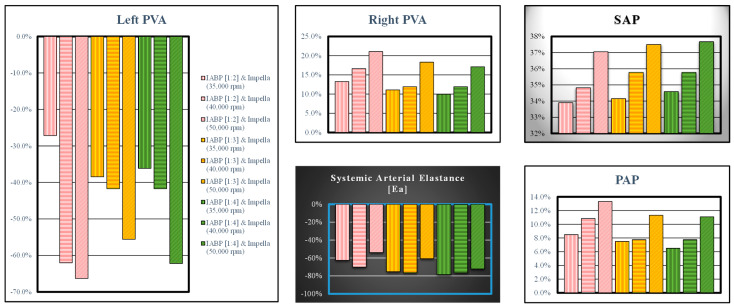
The left (top-middle) panel shows left (right) pressure-volume area; systolic aortic pressure, systemic arterial elastance and pulmonary arterial pressure are available in the top-right, bottom-middle and bottom-right panels, respectively. The bars with vertical (horizontal) lines indicate the percentage change of a variable when the LVAD rotational speed was set to 35,000 (40,000) rpm, while the bars with oblique lines were obtained for a rotational speed of 50,000 rpm. The simulation was performed by setting the IABP in 1:2 (white bars with red lines), 1:3 (yellow bars with red lines) and 1:4 (green bars with black lines) modes.

The combined action of the two devices produced opposite effects on the left ventricular pressure-volume area (left panel in Figure 9) and the right PVA (top-middle panel). In both cases, the highest percentage variations occurred when the LVAD was set to 50,000 rpm. The effects induced on the systemic arterial pressure and systemic arterial elastance were opposite to each other, but in both cases, they were higher when the IABP was activated in the 1:2 mode (top-right and bottom-middle panels in Figure 9, respectively).

## 4. Discussion

Acute myocardial infarction, acute decompensated heart failure and post-cardiotomy failure are the common scenarios leading to cardiogenic shock. The aim is timing of interventions to stabilise the condition and buy time for the next step. The IABP and Impella 2.5 are commonly available in acute settings, either in theatres or in the catheterisation laboratory, where they are mainly used as standalone devices. Their combined use was previously proposed based on experimental evidence [38]. Our simulations show that the highest level of left ventricular unloading is achieved with the combined use of the Impella device at 50,000 rpm and the IABP activated in the 1:2 mode (Figure 9). The Impella device seems to play a major role. The same setting provides a significant increase in coronary blood flow and left ventricular ejection fraction (Figure 8). When the IABP is fully activated (1:1), the highest effect is again achieved with the Impella device at 50,000 rpm (Figure 6 and Figure 7). The outcome of our simulations confirms that the combined use of the two devices seems to achieve a synergistic effect in terms of left ventricular unloading, organ perfusion with particular reference to coronary and cerebral blood flow and oxygen demand/supply. This seems to match the experimental setting [38]. These findings suggest that the combined use of IABP and the Impella device may play a key role in acute settings in terms of the stabilisation of cardiogenic shock, with a view to upgrading to a more advanced device should the escalation of treatment be required. This would allow sufficient time for transfer to a transplant centre to carry out the procedure. Although the 1:4 mode for IABP is not used in clinical practice, it has been included for comparison purposes. The degree of assistance may be tailored according to the group of patients and the scenarios faced. A simulation approach may be of value in terms of clinical decision making. Although little is known about long-term outcomes beyond the first 30 days, this should not be a deterrent but, rather, a motivation to push the boundaries and find solutions to address this problem. Undoubtedly, there remains an ongoing elevated mortality risk even in surviving patients, who require long-term follow-up [39]. Additionally, morbidity in terms of the increased need for home support or transition to long-term care may generate discussions about patients’ willingness to undergo risky interventions, which may improve survival but increase dependence. Teams caring for patients with CS need to recognise this sustained, ongoing risk and further review their approach to ensure sustained, ongoing care. High-intensity follow-up, aggressive medical treatment and device-based intervention for heart failure may help to modulate risk when failure occurs in an acute CS setting. The outcome of our simulations gives a valid theoretical background to the experimental findings and largely contrasts the questionable outcome of the IMPELLA-STIC study [40], in view of its significant limitations. This may help to alleviate the current controversy and support the argument for the potential superiority of the combined use of the Impella device and IABP in acute settings with relatively low mortality, despite the high-risk features of these patients [1]. The selection criteria remain key aspects in terms of suitability and potential benefits [41]. The IABP and Impella remain short-term circulatory support devices, with specific advantages and limitations [2,9]. Nevertheless, their use can be of significant value in acute settings in terms of haemodynamic stabilisation, with a view to recovery and weaning off the device, or escalation to a more advanced form of circulatory support in a tertiary referral transplant centre. The development of expertise in selected and strategically placed non-transplant centres may be more affordable compared to the amount of resources required for additional, highly specialised units. This would potentially give a different direction to the management of cardiogenic shock in terms of case load, skill retention and outcomes based on close cooperation between centres. Clinical trials on cardiogenic shock are difficult to conduct due to multiple factors and may lead to premature termination, inconclusive results and a lack of value [42]. A simulation approach combined with clinical observation may address the problem through a different perspective.

## 5. Conclusions

Mortality and morbidity in cardiogenic shock remain high despite efforts aimed at improving in-hospital or 30-day survival through revascularisation, the use of mechanical circulatory support and medical treatment optimisation. A focus on early intervention to prevent further deterioration may have a role to play. This may be achievable if designated non-transplant centres are available to offer the necessary expertise. Our simulations show that the combination of IABP and the Impella device offers sufficient support to address the acute phase and allow transfer to a transplant centre should escalation to more advanced support be required. Close co-operation between clinicians and engineering scientists may help with treatment optimisation and outcome prediction for different groups of patients.

## Data Availability

The data presented in this study are available on request from the corresponding author.

## References

[1] Bhuiyan R., Bimal T., Fishbein J., Gandotra P., Selim S., Ong L., Gruberg L. (2023). Percutaneous coronary intervention with Impella support with and without intra-aortic balloon in cardiogenic shock patients. Cardiovasc. Revascularization Med..

[2] Wong A.S.K., Sin S.W.C. (2020). Short-term mechanical circulatory support (intra-aortic balloon pump, Impella, extracorporeal membrane oxygenation, TandemHeart): A review. Ann. Transl. Med..

[3] Merdji H., Levy B., Jung C., Ince C., Siegemund M., Meziani F. (2023). Microcirculatory dysfunction in cardiogenic shock. Ann. Intensiv. Care.

[4] De Lazzari B., Capoccia M., Badagliacca R., Bozkurt S., De Lazzari C. (2023). IABP versus Impella Support in Cardiogenic Shock: “In Silico” Study. J. Cardiovasc. Dev. Dis..

[5] De Lazzari B., Iacovoni A., Mottaghy K., Capoccia M., Badagliacca R., Vizza C.D., De Lazzari C. (2021). ECMO Assistance during Mechanical Ventilation: Effects Induced on Energetic and Haemodynamic Variables. Comput. Methods Programs Biomed..

[6] Saito S., Shibasaki I., Matsuoka T., Niitsuma K., Hirota S., Kanno Y., Kanazawa Y., Tezuka M., Takei Y., Tsuchiya G. (2022). Impella support as a bridge to heart surgery in patients with cardiogenic shock. Interact. Cardiovasc. Thorac. Surg..

[7] Ezad S.M., Ryan M., Donker D.W., Pappalardo F., Barrett N., Camporota L., Price S., Kapur N.K., Perera D. (2023). Unloading the Left Ventricle in Venoarterial ECMO: In Whom, When, and How?. Circulation.

[8] Møller J.E., Sionis A., Aissaoui N., Ariza A., Bělohlávek J., De Backer D., Färber G., Gollmann-Tepeköylu C., Mebazaa A., Price S. (2023). Step by step daily management of short-term mechanical circulatory support for cardiogenic shock in adults in the intensive cardiac care unit: A clinical consensus statement of the Association for Acute CardioVascular Care of the European Society of Cardiology SC, the European Society of Intensive Care Medicine, the European branch of the Extracorporeal Life Support Organization, and the European Association for Cardio-Thoracic Surgery. Eur. Heart J. Acute Cardiovasc. Care.

[9] Russo G., Burzotta F., Aurigemma C., Pedicino D., Romagnoli E., Trani C. (2022). Can we have a rationalized selection of intra-aortic balloon pump, Impella, and extracorporeal membrane oxygenation in the catheterization laboratory?. Cardiol. J..

[10] Cavayas Y.A., Noly P.-E., Singh G., Lamarche Y. (2021). Controversies in extracorporeal membrane oxygenation: Immediate versus watchful waiting for venoarterial extracorporeal membrane oxygenation venting. JTCVS Open.

[11] Pavlidis A.N., Redwood S.R., Clapp B.R. (2014). Combined hemodynamic support with the Impella 2.5 device and intra-aortic balloon pump for management of refractory cardiogenic shock. J. Invasive Cardiol..

[12] Gupta A., Allaqaband S., Bajwa T. (2009). Combined use of impella device and intra-aortic balloon pump to improve survival in a patient in profound cardiogenic shock post cardiac arrest. Catheter. Cardiovasc. Interv..

[13] Wiktor D.M., Sawlani N., Kanthi Y., Sipahi I., Fang J.C., Blitz A. (2010). Successful combined use of Impella Recover 2.5 device and intra-aortic balloon pump support in cardiogenic shock from acute myocardial infarction. ASAIO J..

[14] Cubeddu R.J., Lago R., Horvath S.A., Vignola P.A., O’Neill W., Palacios I.F. (2012). Use of the Impella 2.5 system alone, after and in combination with an intra-aortic balloon pump in patients with cardiogenic shock: Case description and review of the literature. EuroIntervention.

[15] Møller-Helgestad O.K., Poulsen C.B., Christiansen E.H., Lassen J.F., Ravn H.B. (2015). Support with intra-aortic balloon pump vs. Impella2.5^®^ and blood flow to the heart, brain and kidneys—An experimental porcine model of ischaemic heart failure. Int. J. Cardiol..

[16] Lansky A.J., Tirziu D., Moses J.W., Pietras C., Ohman E.M., O’Neill W.W., Ekono M.M., Grines C.L., Parise H. (2022). Impella Versus Intra-Aortic Balloon Pump for High-Risk PCI: A Propensity-Adjusted Large-Scale Claims Dataset Analysis. Am. J. Cardiol..

[17] Meani P., Lorusso R., Pappalardo F. (2022). ECPella: Concept, Physiology and Clinical Applications. J. Cardiothorac. Vasc. Anesth..

[18] Weber D.M., Raess D.H., Henriques J., Siess T. (2009). Principles of Impella Cardiac Support. Card. Interv. Today.

[19] De Lazzari B., Badagliacca R., Filomena D., Papa S., Vizza C.D., Capoccia M., De Lazzari C. (2022). CARDIOSIM©: The First Italian Software Platform for Simulation of the Cardiovascular System and Mechanical Circulatory and Ventilatory Support. Bioengineering.

[20] Kaye D.M., Wolsk E., Nanayakkara S., Mariani J., Hassager C., Gustafsson F., Moller J.E., Sunagawa K., Burkhoff D. (2021). Comprehensive Physiological Modeling Provides Novel Insights Into Heart Failure with Preserved Ejection Fraction Physiology. J. Am. Heart Assoc..

[21] De Lazzari B., Iacovoni A., Capoccia M., Papa S., Badagliacca R., Filomena D., De Lazzari C. (2022). Ventricular and Atrial Pressure—Volume Loops: Analysis of the Effects Induced by Right Centrifugal Pump Assistance. Bioengineering.

[22] Manzi G., Miotti C., Mariani M.V., Papa S., Luongo F., Scoccia G., De Lazzari B., De Lazzari C., Benza R.L., Fedele F. (2021). Computational Simulator Models and Invasive Hemodynamic Monitoring as Tools for Precision Medicine in Pulmonary Arterial Hypertension. J. Clin. Med..

[23] Ferrari G., Kozarski M., De Lazzari C., Ska K.G., Tosti G., Darowski M. (2005). Development of a hybrid (numerical-hydraulic) circulatory model: Prototype testing and its response to IABP assistance. Int. J. Artif. Organs.

[24] Kung E., Farahmand M., Gupta A. (2019). A Hybrid Experimental-Computational Modeling Framework for Cardiovascular Device Testing. J. Biomech. Eng..

[25] Bozkurt S., Paracha W., Bakaya K., Schievano S. (2022). Patient-Specific Modelling and Parameter Optimisation to Simulate Dilated Cardiomyopathy in Children. Cardiovasc. Eng. Technol..

[26] De Lazzari C., Stalteri D. 2011–2019, CARDIOSIM© Website. https://cardiosim.dsb.cnr.it.

[27] Vahdatpour C., Collins D., Goldberg S. (2019). Cardiogenic Shock. J. Am. Heart Assoc..

[28] Ng P.Y., Ma T.S.K., Ip A., Fang S., Li A.C.C., Wong A.S.K., Ngai C.W., Chan W.M., Sin W.C. (2023). Effects of varying blood flow rate during peripheral veno-arterial extracorporeal membrane oxygen (V-A ECMO) on left ventricular function measured by two-dimensional strain. Front. Cardiovasc. Med..

[29] Caldas J.R., Panerai R.B., Bor-Seng-Shu E., Almeida J.P., Ferreira G.S.R., Camara L., Nogueira R.C., Oliveira M.L., Jatene F.B., Robinson T.G. (2017). Cerebral hemodynamics with intra-aortic balloon pump: Business as usual?. Physiol. Meas..

[30] Alkan R., De Lazzari B., Capoccia M., De Lazzari C., Bozkurt S. (2023). Computational Evaluation of IABP, Impella 2.5, TandemHeart and Combined IABP and Impella 2.5 Support in Cardiogenic Shock. Mathematics.

[31] De Lazzari C. (2012). Interaction between the septum and the left (right) ventricular free wall in order to evaluate the effects on coronary blood flow: Numerical simulation. Comput. Methods Biomech. Biomed. Eng..

[32] Capoccia M., Marconi S., De Lazzari C. (2018). Decision-making in advanced heart failure patients requiring LVAD insertion: Can preoperative simulation become the way forward? A case study. J. Biomed. Eng. Inform..

[33] Schurtz G., Rousse N., Saura O., Balmette V., Vincent F., Lamblin N., Porouchani S., Verdier B., Puymirat E., Robin E. (2021). IMPELLA^®^ or Extracorporeal Membrane Oxygenation for Left Ventricular Dominant Refractory Cardiogenic Shock. J. Clin. Med..

[34] Martinho S., Adão R., Leite-Moreira A.F., Brás-Silva C. (2020). Persistent Pulmonary Hypertension of the Newborn: Pathophysiological Mechanisms and Novel Therapeutic Approaches. Front. Pediatr..

[35] Abramov D., Haglund N.A., Di Salvo T.G. (2017). Effect of Milrinone Infusion on Pulmonary Vasculature and Stroke Work Indices: A Single-Center Retrospective Analysis in 69 Patients Awaiting Cardiac Transplantation. Am. J. Cardiovasc. Drugs.

[36] Kosaraju A., Pendela V.S., Hai O. (2023). Cardiogenic Shock. StatPearls.

[37] van Diepen S., Katz J.N., Albert N.M., Henry T.D., Jacobs A.K., Kapur N.K., Kilic A., Menon V., Ohman E.M., Sweitzer N.K. (2017). Contemporary Management of Cardiogenic Shock: A Scientific Statement from the American Heart Association. Circulation.

[38] Sauren L.D.C., Accord R.E., Hamzeh K., de Jong M., van der Nagel T., van der Veen F.H., Maessen J.G. (2007). Combined Impella and Intra-aortic Balloon Pump Support to Improve Both Ventricular Unloading and Coronary Blood Flow for Myocardial Recovery: An Experimental Study. Artif. Organs.

[39] Sterling L.H., Fernando S.M., Talarico R., Qureshi D., van Diepen S., Herridge M.S., Price S., Brodie D., Fan E., Di Santo P. (2023). Long-Term Outcomes of Cardiogenic Shock Complicating Myocardial Infarction. J. Am. Coll. Cardiol..

[40] Bochaton T., Huot L., Elbaz M., Delmas C., Aissaoui N., Farhat F., Mewton N., Bonnefoy E. (2020). Mechanical circulatory support with the Impella^®^ LP5.0 pump and an intra-aortic balloon pump for cardiogenic shock in acute myocardial infarction: The IMPELLA-STIC randomi.sed study. Arch. Cardiovasc. Dis..

[41] Rohm C.L., Gadidov B., Leitson M., Ray H.E., Prasad R. (2019). Predictors of Mortality and Outcomes of Acute Severe Cardiogenic Shock Treated with the Impella Device. Am. J. Cardiol..

[42] Ouweneel D.M., Engstrom A.E., Sjauw K.D., Hirsch A., Hill J.M., Gockel B., Tuseth V., van der Schaaf R.J., Henriques J.P.S. (2016). Experience from a randomised controlled trial with Impella 2.5 versus IABP in STEMI patients with cardiogenic pre-shock. Lessons learnt from the IMPRESS in STEMI trial. Int. J. Cardiol..

